# Combining the Burrows-Wheeler Transform and RCM-LDGM Codes for the Transmission of Sources with Memory at High Spectral Efficiencies

**DOI:** 10.3390/e21040378

**Published:** 2019-04-08

**Authors:** Imanol Granada, Pedro M. Crespo, Javier Garcia-Frías

**Affiliations:** 1 Department of Basic Science, Tecnun—University of Navarra, 20018 San Sebastian, Spain; 2 Department of Electrical and Computer Engineering, University of Delaware, Newark, DE 19716, USA

**Keywords:** Burrows-Wheeler Transform, hidden Markov models, Markov processes, rate adaptation, RCM-LDGM codes

## Abstract

In this paper, we look at the problem of implementing high-throughput Joint Source- Channel (JSC) coding schemes for the transmission of binary sources with memory over AWGN channels. The sources are modeled either by a Markov chain (MC) or a hidden Markov model (HMM). We propose a coding scheme based on the Burrows-Wheeler Transform (BWT) and the parallel concatenation of Rate-Compatible Modulation and Low-Density Generator Matrix (RCM-LDGM) codes. The proposed scheme uses the BWT to convert the original source with memory into a set of independent non-uniform Discrete Memoryless (DMS) binary sources, which are then separately encoded, with optimal rates, using RCM-LDGM codes.

## 1. Introduction

When considering sources with memory, Shannon’s JSC coding theorem states that reliable transmission is only possible if

(1) ℋ(S)R≤C,

where ℋ is the entropy rate of the source in bits per source symbol, *C* is the capacity of the channel in information bits per channel use and *R* is the JSC code’s rate (source symbols per channel use). A fundamental result of information theory is the Separation Theorem, which states that provided unbounded delay, no optimality is lost by designing the joint decoder as the concatenation of an optimal source code with compression rate ℋ, and a capacity-achieving channel code of rate *C*. This independent design of source and channel codes allows for diverse sources to share the same digital media. Therefore, source coding and channel coding have traditionally been addressed independently of each other.

Nevertheless, when complexity is an issue and the length of the input block is constrained, the overall performance can be improved by using a JSC coding scheme. In this case, the joint decoder employs the inherent redundancy of the uncompressed source [1]. The main approaches to JSC can be categorized as follows:

- Ad hoc approaches where the channel encoder is applied to a given source compression format [2,3,4]. High-level information from the source code is used in the decoding process, which makes this approach highly dependent on the source encoder.
- Schemes where, assuming a Markovian model for the source [5,6], the factor graph of the source is combined with the one representing the channel code in order to perform joint decoding, such as in [7,8,9] (using Turbo codes) or in [10,11] (using LDPC codes).
- Strategies where context estimation techniques such as the Discrete Universal Denoiser (DUDE) [12] or the Burrow-Wheeler Transform [13,14,15] are used to help the decoding process.

To the best of our knowledge, when considering discrete sources with memory, existing JSC schemes in the literature [7,8,9,10,11,12,13,14,15,16] are restricted to encoders that produce binary output symbols, such as Turbo, LDPC, or Polar codes. This leads to throughputs (or spectral efficiencies) bounded by 2 binary source symbols per complex channel use. To achieve higher rates, larger alphabets must be used for the encoded symbols. To that end, we will consider a high-throughput, hybrid analog-digital JSC coding scheme, recently proposed for non-uniform discrete memoryless sources (DMSs) in [17,18]. This hybrid scheme is constructed by generating most of the output symbols from weighted linear combinations of the input bits, as in Rate-Compatible Modulation (RCM), and a few of them using a Low-Density Generator Matrix (LDGM) code. The basic idea is that the RCM scheme corrects most of errors, leaving residual errors to be corrected by the LDGM code. Source non-uniformity is exploited at the decoder and the encoder is optimized depending on the source non-uniformity to further improve the system performance. In what follows we will denote these codes by RCM-LDGM. These codes provide smooth rate adaptation in a broad dynamic range in a very simple way, adding or removing rows to the encoding matrix.

The proposed high-throughput JSC scheme belongs to the third group of techniques. It uses the BWT to convert the original source with memory into a set of independent non-uniform discrete (binary) memoryless sources (DMSs). The resulting DMSs are each RCM-LDGM encoded with rates adapted to the entropy of the corresponding DMS. It should be pointed out that the authors in [13,14] use the BWT as a context estimation tool to help in the iterative decoding process. Specifically, they apply the BWT at each iterative decoding step and then pass the first order probability distribution of its output to the constituent decoders of the LDPC or Turbo codes. Differently, and different from [15], which applies the BWT in the transmitter before coding and then uses the first order probability distribution of the BWT output sequences to optimize the output energy of the binary modulator, the proposed scheme uses this first order probability distribution to optimize the rates at which different segments of the BWT output sequences are transmitted. Thus, the main contribution of this paper is the proposal of a novel high-throughput JSC scheme for sources with memory based on the application of the BWT and optimal rate allocation. To the best of our knowledge, for sources with memory no high-throughput JSC system has appeared in the literature.

The remainder of this paper is organized as follows. Section 2 briefly reviews some preliminary concepts required for the explanation of the proposed BWT-JSC scheme. Section 3 presents our proposed JSC scheme, leaving for Section 4 the corresponding performance evaluation. Finally, Section 5 provides the concluding remarks.

## 2. Preliminaries

In this section, we briefly review the statistical characterization of a binary source with memory by Markov Models, the Burrows-Wheeler Transform, and the design of parallel RCM-LDGM codes, which are the building blocks of our proposed BWT-JSC scheme.

### 2.1. Markov Sources

We consider binary sources with memory such that the stationary output sequence follows a time-invariant, HMM with λ states $\left\{S_{1},\ldots ,S_{\lambda }\right\}$. We denote the states of the source at time *k* as $q_{k}$. Complete specification of a binary HHM requires the specification of three probability measures, *A*, *B*, and π, defined as:

- $A=[a_{ij}]$ is the state transition probability matrix of dimension λ×λ, with $a_{ij}$ the probability of transition from state $S_{i}$ to state $S_{j}$, i.e., $a_{i,j}=P\left(q_{k+1}=S_{j}|q_{k}=S_{i}\right)$ for all *k*.
- $B=[b_{j}\left(v\right)]$ is the observation symbol probability matrix, with $b_{j}\left(v\right)$ the probability of getting in the binary symbol *v* in state $S_{j}$, i.e., $b_{j}\left(v\right)=P\left(v|S_{j}\right)$, 1≤j≤λ, v∈{0,1}.
- π is the initial state distribution vector, with $\pi_{j}$ the probability for the initial state to be $S_{j}$, i.e., $\pi_{j}=P\left(q_{1}=S_{j}\right)$, 1≤j≤λ.

**Remark** **1.** *For stationary sources, π should be taken as the stationary distribution of the chain, i.e.,*π=Aπ.

**Remark** **2.** *When matrix B has entries 0 and 1, the HMM reduces to a MC.*

### 2.2. Burrows-Wheeler Transform (BWT)

The BWT [19] is a lexicographical permutation of the characters of a string such that the transformed sequence is easier to be compressed. It is obtained from the last column of an array whose rows are all cyclic shifts from the input in dictionary order, which tend to have long runs of identical characters. From this last string we can recover the entire array, making the BWT reversible. The BWT has been widely analyzed in [20,21,22] and employed for the general problem of data compression [23,24]. More recent contributions have focused on the applicability of the BWT to coded transmission of Markov sources through AWGN channels via LDPC [13] and non-systematic Turbo codes [14].

Let $T={\left\{T_{k}\right\}}_{k=1}^{K}$, $T_{k}\in \left\{0,1\right\}$ denote the output block of the reversible block-sorting BWT when its input is the block of binary source symbols ${\left\{U_{k}\right\}}_{k=1}^{K}$. For sources modeled by MCs with λ states, it was shown in [20] that the joint probability mass function, $P_{T}\left(t\right)$, of the random block T is approximately memoryless and piecewise stationary, in the sense that there exist λ index sets, $L_{i}=\left\{w_{i-1}\ldots w_{i}\right\}$, i=1,…,λ with $w_{0}=1$ and $w_{\lambda }=K+1$, and a probability distribution

(2) $$Q_{T}\left(t\right)=\prod_{i=1}^{\lambda }\prod_{k=w_{i-1}}^{w_{i}-1}Q_{i}\left(t_{k}\right)$$

such that the normalized divergence between both distributions can be made arbitrarily small for sufficiently large *K*, i.e.,

(3) $$\frac{1}{K}D\left(Q_{T}\left(t\right)∥P_{T}\left(t\right)\right)\to 0$$

as K→∞.

As the block length *K* goes to infinity, the normalized length of the index set in expression (2) converges to $c_{i}\in R$, i.e., $\lim_{K\to \infty }\frac{|L_{i}|}{K}=c_{i}$.

**Definition** **1.** *Let*$T_{i}$*denote the binary random sequence of length*$K_{i}=c_{i}K$*at the output of the BWT corresponding to the index set*$L_{i}$*,*i=1,…,λ*. That is,*$T_{i}={\left\{T_{k}\right\}}_{k\in L_{i}}$.

Observe from (2) that for large blocks of length *K*, the binary random symbols $T_{k}\in T_{i}$, with $k\in L_{i}$, can be considered independent and identical distributed (i.i.d.), with probability distribution

(4) $$\begin{array}{c} Q_{i}\left(t_{k}\right)≜\left\{\begin{array}{cc} p_{i}^{0} & \mathrm{if}\;t_{k}=0\\ p_{i}^{1}=1-p_{i}^{0} & \mathrm{if}\;t_{k}=1 \end{array}\right\} \end{array}$$

for some $p_{i}^{0}\in \left(0,1\right)$. These approximations should be understood under the convergence criterium (3).

Therefore, we will model the non-stationary BWT output sequence T as the concatenation of λ blocks of length $K_{i}=c_{i}K$, i=1,…,λ generated by λ independent DMS binary sources $S_{1},S_{2}\ldots ,S_{\lambda }$, with entropies

$$H_{i}=-p_{i}^{0}\log p_{i}^{0}-\left(1-p_{i}^{0}\right)\log\left(1-p_{i}^{0}\right),\;\;i=1,2,\ldots ,\lambda .$$

By the independence of the sources and their symbols, the entropy rate of the original source can be expressed as

(5) $$ℋ\left(S\right)=\sum_{i=1}^{\lambda }\frac{K_{i}}{K}H_{i}=\sum_{i=1}^{\lambda }c_{i}H_{i}.$$

### 2.3. Parallel RCM-LDGM Codes

The *N*-length codeword of a parallel concatenation of RCM and LDGM x, is composed of *M* RCM coded symbols and I=N−M LDGM coded bits. Next, we provide a succinct overview of the constituent RCM and LDGM codes.

#### 2.3.1. Rate-Compatible Modulation (RCM) Codes

RCM codes [25] are based on random projections which generate multilevel symbols from weighted linear combinations of the source binary symbols. More precisely, an RCM code of rate K/M is generated by an M×K sparse mapping matrix *G*. The non-zero entries of each row of *G* belong to a multiset ±D, with D⊂N, the set of natural numbers (positive integers). Given the binary source sequence $u=\{u_{1},u_{2},\ldots ,u_{K}\}$, the RCM coded sequence c of length *M* is obtained as

$$c=[c_{1},c_{2},\ldots ,c_{M}]=Gu$$

where these operations are in the real field. Finally, rate adaptation is achieved by adjusting the number of rows in *G*.

#### 2.3.2. Low-Density Generator Matrix (LDGM) Codes

LDGM codes are a subclass of the well-known LDPC codes with the particularity that the generator matrix $G_{L}$ is also sparse. This allows the decoding algorithm to use the graph generated by $G_{L}$. In this paper, we consider systematic LDGM codes, whose generator matrix is of the form $G_{L}=\left[I_{K}|P\right]$, where $I_{K}$ is the identity matrix of size *K* and *P* is a regular K×I sparse matrix with $d_{LDGM}^{\left(v\right)}$ non-zero elements in each column. The LDGM coded sequence c of length N=K+I is obtained as

$$c=\left[c_{1},c_{2},\ldots ,c_{N}\right]=u^{⊺}G_{L}=u^{⊺}\left[I_{K}|P\right]=\left[u_{1},u_{2},\ldots ,u_{K},x_{1},x_{2},\ldots ,x_{I}\right],$$

where $u=\{u_{1},u_{2},\ldots ,u_{K}\}$ is the binary source sequence to be transmitted and the operations are in the binary field. Unlike general LDPC codes, LDGM codes suffer from high error floor [26]. However, it has been shown that they can help to lower the error floor of other codes as explained next.

#### 2.3.3. Parallel RCM-LDGM Code

Consider an RCM code of rate K/M generated by a matrix *G*, and the non-systematic part of a high rate binary regular LDGM of rate K/I, generated by *P*. Then, the parallel RCM-LDGM coded sequence x of length M+I is given by

$$x^{⊺}=\left[{\left(Gu\right)}^{⊺}\;|\;2\cdot \left(\left(u^{⊺}P\;\mathrm{mod}\;2\right)-\frac{1}{2}\right)\right],$$

where the last *I* symbols are encoded using a BPSK modulator. Recall that the objective of the LDGM code is to correct the residual error of the RCM code, lowering the error floor but without degrading the RCM waterfall region.

Finally, the coded symbols of x are grouped two by two and transmitted using a QAM modulator, so that the spectral efficiency, ρ, is

$$\rho =\frac{2\cdot K}{M+I}$$

binary source symbols per complex channel use.

**Figure 1:**
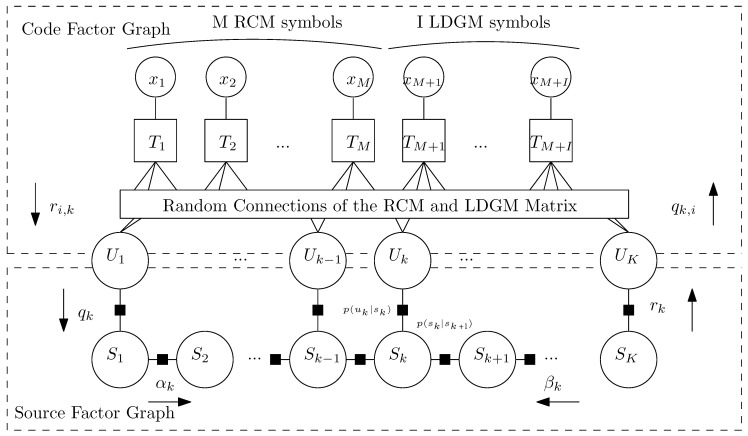
Factor graph of the parallel RCM-LDGM code incorporating the factor graph modeling the source.

The performance of RCM-LDGM codes when encoding uniform and non-uniform DMSs can be found in [17,18]. An efficient way to design these codes was shown in [27]. However, no results have been found in the literature regarding the use of parallel RCM-LDGM codes to encode discrete binary sources with memory. The conventional approach in this situation would be to encode the correlated source symbols at the transmitter by the RCM-LDGM encoder, and to modify the decoder at the receiver to exploit the correlation of the source. This may be done by incorporating the factor graph that models the source into the factor graph of the RCM-LDGM code, and running the sum-product algorithm [28] over the whole factor graph represented in Figure 1. We will denote this approach as NON-BWT-JSC, and we will compare it with our proposed coding scheme defined in the next section.

## 3. Proposed BTW-JSC Scheme

The main idea behind the proposed BWT-JSC scheme is to transform the original source with memory *S*, into a set of λ independent non-uniform memoryless binary sources. This is accomplished by partitioning the source sequence into blocks of length *K*, $U^{\left(l\right)}={\left\{U_{l\cdot K+k}\right\}}_{k=1}^{K}$, l∈N, and then applying the BWT to each of these blocks. The corresponding output segment *i*, inside output block *l*, is given by

$$T_{i}^{\left(l\right)}={\left\{T_{l\cdot K+k}\right\}}_{k=w_{i-1}}^{w_{i}}.$$

**Figure 2:**
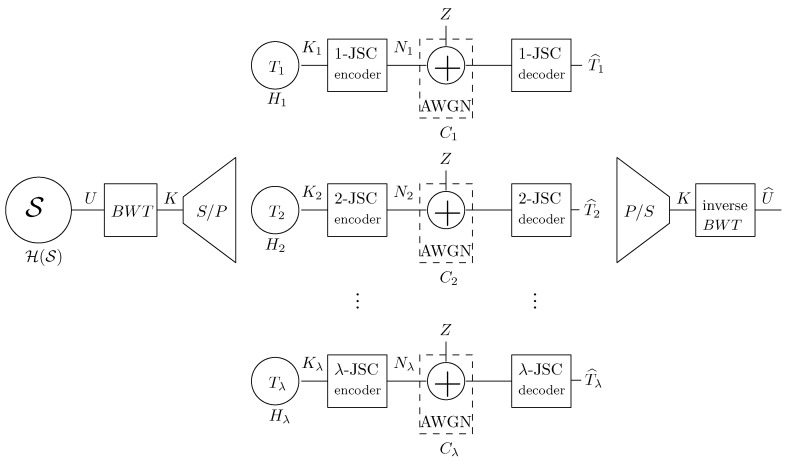
BWT-based proposed communication system. Please note that $K=\sum_{i=1}^{\lambda }T_{i}$.

Observe that the sequence blocks $T_{i}^{\left(l\right)}$, i=0,1⋯,λ can be considered to have been generated by a non-uniform DMS with entropy $H_{i}$, i=1,2,…,λ. Therefore, we have reduced the encoding problem of sources with memory to a simpler one, namely the problem of JSC coding of non-uniform *memoryless* binary sources, with entropies $H_{i}$. Notice that the previously mentioned RCM-LDGM high-throughput, JSC codes for non-uniform DMS sources [17], can now be applied to each of the λ independent sources as shown in Figure 2.

More concretely, let us consider a source with memory, S, and with entropy rate ℋ(S), which generates blocks of *K* binary symbols to be transmitted at rate R=K/N by the parallel JSC coding system of Figure 2. Let $T_{i}$ (refer to Definition 1) be the input sequence to the corresponding *i*-JSC code of rate $R_{i}=K_{i}/N_{i}$, under the constraint $N=\sum_{i=1}^{\lambda }N_{i}$. Denote by ${\left\{SNR_{i}\right\}}_{i=1}^{\lambda }$ the set of signal-to-noise ratios allocated to each parallel channel. Define by

$$\bar{SNR}=\sum_{i=1}^{\lambda }\frac{N_{i}}{N}SNR_{i}$$

the average SNR over all parallel channels. The following Theorem proves that the proposed scheme achieves the Shannon limit.

**Theorem** **1.** *Given a target rate R, the minimum overall*$\bar{SNR}$*in the coding scheme of Figure 2 is achieved when all the*$SNR_{i}$*’s take the same value, given by the SNR Shannon limit from expression (1), i.e.,*$SNR_{i}^{*}=2^{Rℋ(S)}-1$*. The individual rates*$R_{i}$*are given by*$R_{i}=\frac{Rℋ\left(S\right)}{H_{i}}$, i=1,…,λ.

**Proof.**  Given a set of signal-to-noise ratios ${\left\{{\mathrm{SNR}}_{i}\right\}}_{i=1}^{\lambda }$, the rates of the JSC encoders in Figure 2 are given by the Shannon’s separation theorem as

$$R_{i}=\frac{K_{i}}{N_{i}}=\frac{C({\mathrm{SNR}}_{i})}{H_{i}},\;i=1,\ldots ,\lambda ,$$

where by the BWT hypothesis, $K=\sum_{i=1}^{\lambda }K_{i}$.

We seek to minimize the average signal-to-noise ratio $\bar{SNR}$ over all the λ parallel AWGN channels, i.e.,

$$\begin{array}{c} \bar{SNR}=\sum_{i=1}^{\lambda }\frac{N_{i}}{\sum_{j=1}^{\lambda }N_{j}}{\mathrm{SNR}}_{i}=\sum_{i=1}^{\lambda }\frac{K_{i}}{N}\frac{H_{i}}{C({\mathrm{SNR}}_{i})}SNR_{i}, \end{array}$$

under the constraint of achieving a rate

$$R=\frac{K}{N}=\sum_{i=1}^{\lambda }\frac{K_{i}}{\sum_{j=1}^{\lambda }N_{j}}.$$

Please note that since $K=\sum_{i=1}^{\lambda }K_{i}$ is fixed, the constraint in *R* reduces to the constraint

(6) $$N=\sum_{j=1}^{\lambda }N_{j}=\sum_{j=1}^{\lambda }\frac{H_{j}K_{j}}{C({\mathrm{SNR}}_{j})}.$$

By applying the Lagrange multipliers method, we define *F* as

$$\begin{array}{c} F=\sum_{i=1}^{\lambda }\frac{K_{i}H_{i}}{N}\frac{{\mathrm{SNR}}_{i}}{C\left({\mathrm{SNR}}_{i}\right)}+\gamma \left(\sum_{i=1}^{\lambda }\frac{K_{i}H_{i}}{C\left({\mathrm{SNR}}_{i}\right)}-N\right), \end{array}$$

and by searching for an extreme of *F*, we obtain that the optimal ${\mathrm{SNR}}_{i}^{*}$ are all equal to some value Γ. Therefore, from constraint (6)

$$\begin{array}{c} N=\sum_{i=1}^{\lambda }N_{i}=\sum_{i}\frac{K_{i}H_{i}}{C\left(\Gamma \right)}=\frac{Kℋ\left(S\right)}{C\left(\Gamma \right)}, \end{array}$$

where the last equality follows from expression (5). Thus, the rate can be written as

$$R=\frac{K}{N}=\frac{C(\Gamma )}{ℋ\left(S\right)}.$$

Consequently, the value of Γ is given by the signal-to-noise ratio required to achieve the same rate *R* in the standard point-to-point communications system. That is,

$$\Gamma =2^{RH(S)}-1.$$

We conclude that

$${\bar{SNR}}^{*}=SNR_{i}^{*}=2^{Rℋ(S)}-1$$

and

(7) $$\begin{array}{c} R_{i}=\frac{K_{i}}{N_{i}}=\frac{C({\bar{SNR}}^{*})}{H_{i}}=\frac{Rℋ\left(S\right)}{H_{i}}. \end{array}$$

 □

**Remark** **3.** *Observe that the BWT-JSC is asymptotically optimal in the sense that can achieve the SNR Shannon limit given by the Separation Theorem.*

## 4. Results

In this section, we evaluate the proposed scheme, comparing its performance with the conventional NON-BWT-JSC approach described in Section 2.3, which is based on a single code. Without any loss of generality, the spectral efficiency of the communication system has been set to 7.4 binary source symbols per complex channel use, and the source block length to K= 37,000. Thus, the total number of coded symbols at the output of the JSC encoder is N= 10,000. We begin by specifying the Markov sources used in the simulations.

### 4.1. Simulated Sources and Their Output Probability Profile

Three different 2-state (λ=2) Markov sources have been chosen. Two are modeled by MCs, with entropy rates 0.57 and 0.80 bits per source symbol, whereas the third is modeled by a HMM with entropy rate 0.73. For the sake of notation, they will be referred as $S_{1}$, $S_{2}$ and $S_{3}$. Table 1 summarizes their corresponding Markov parameters.

**Figure 3:**
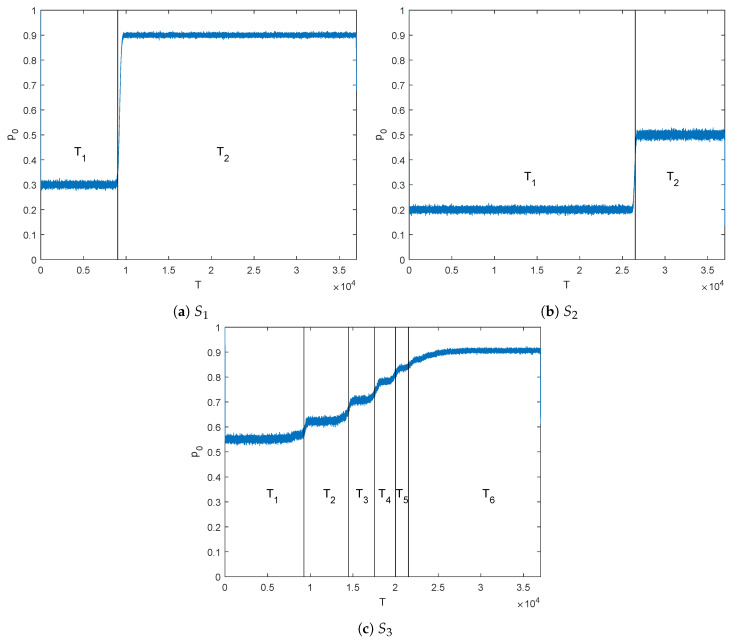
First order probability profiles of the output blocks of the BWT for example sources (**a**) $S_{1}$, (**b**) $S_{2}$ and (**c**) $S_{3}$.

Figure 3 shows the probability mass function $P_{T}\left(t\right)$ (refer to (4)) of the binary random block T of length K= 37,000 at the output of the BWT for sources $S_{1}$, $S_{2}$, and $S_{3}$. Observe that due to the fact that sources $S_{1}$ and $S_{2}$ follow a 2-state MC behavior, the BWT will produce approximately two i.i.d. segment $T_{1}$ and $T_{2}$. This is clearly shown in Figure 3a,b, with segments of length ($K_{1}=9020,\;K_{2}=$ 27,980) with first order probabilities ($p_{0}^{\left(1\right)}\approx 0.3,\;p_{0}^{\left(2\right)}\approx 0.9$) for $S_{1}$ and ($K_{1}=9020,\;K_{2}=$ 27,980) with probabilities ($p_{0}^{\left(1\right)}\approx 0.2,\;p_{0}^{\left(2\right)}\approx 0.5$) for $S_{2}$. On the contrary, the source $S_{3}$ is characterized by a 2-state hidden Markov model, and the hidden property has the effect of increasing the number of states, should the HMM source be approximated by a pure MC. This is observed in Figure 3c, where a 6-state MC source will fairly approximate the statistics of source $S_{3}$. The partition into 6 segments has been decided by the authors based on significant change in the a priori probability of the bits forming the segments. In this case, the first order probabilities of segments $T_{1}-T_{6}$ of sizes ($K_{1}=9250,\;K_{2}=5250,\;K_{3}=3000,\;K_{4}=2500,\;K_{5}=1500,\;K_{6}=$ 15,500) are given by $p_{0}^{\left(1\right)}\approx 0.55,\;p_{0}^{\left(2\right)}\approx 0.63,\;p_{0}^{\left(3\right)}\approx 0.71,\;p_{0}^{\left(4\right)}\approx 0.78,\;p_{0}^{\left(5\right)}\approx 0.84$ and $\;p_{0}^{\left(6\right)}\approx 0.9$.

### 4.2. Numerical Results

In this section, we present the results obtained by Monte Carlo simulation for the proposed BWT-JSC and the conventional NON-BWT-JSC coding schemes. Observe that due to the BWT block, in our proposed scheme a single error at the output of the decoders will be propagated after applying the inverse-BWT. Therefore, to make a fair comparison, the results are presented in the form of Packet Error Rate (PER) versus SNR. It should be mentioned that for the correct recovery of the original transmitted source block, the inverse-BWT at the receiver side needs to know the exact position where the original End of File symbol has been moved by the BWT at the transmitted side. Therefore, this additional information should also be transmitted. Please note that for a 37,000 block length, this position can be addressed by adding 16 binary symbols. In this work, we have considered this rate loss as negligible, but in real scenarios it must be taken into account.

**Figure 4:**
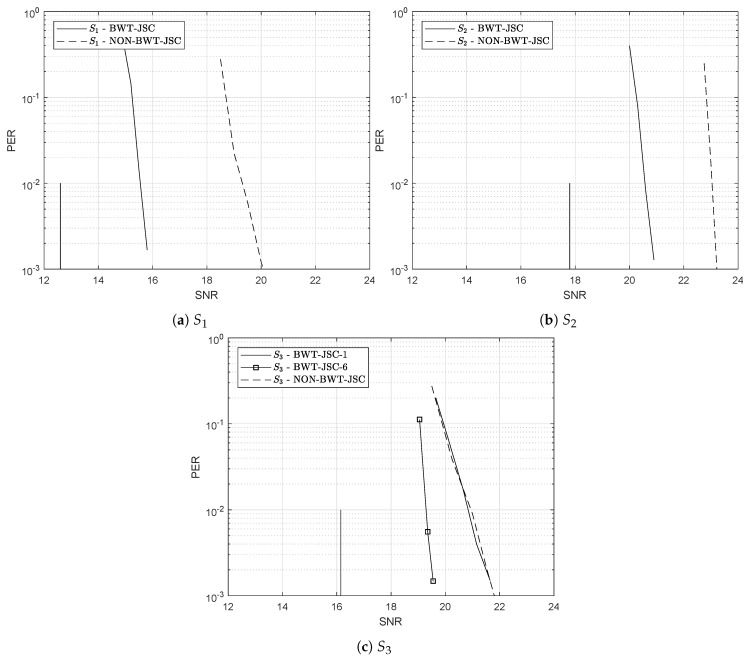
Obtained PER vs SNR curves for the NON-JSC-BWT and JSC-BWT schemes when sources (**a**) $S_{1}$; (**b**) $S_{2}$ and (**c**) $S_{3}$ are considered. The corresponding Shannon limits are plotted in vertical lines.

Figure 4 shows the PER vs SNR curves obtained by simulations for the example sources (**a**) $S_{1}$, (**b**) $S_{2}$ and (**c**) $S_{3}$ when using both the proposed system (BWT-JSC) and the conventional approach (NON-BWT-JSC) as a reference. In the proposed scheme, as stated in Section 3, after performing the BWT, each of the resulting λ independent non-uniform i.i.d. segments $T_{i}\left(p_{0}^{\left(i\right)}\right)$ i=1,…λ (refer to Figure 3), are encoded by λ separated RCM-LDGM JSC codes of rates $R_{i}$ as given by Theorem 1. The codes used for each DMS in the BWT-JSC approach, as well as the one used in the conventional NON-BWT-JSC scheme are summarized in Table 2.

Observe from Figure 4a,b that for sources $S_{1}$ and $S_{2}$, represented by a MC, our BWT-JSC scheme outperforms the NON-BWT-JSC approach by about 4.2 and 2.3 dB’s, respectively. The reason behind this improvement lies in the fact that in the NON-BWT-JSC system, the Factor Graph (FG) of the decoder, results from a parallel concatenation of two sub-graphs: The RCM-LDGM code and MC source sub-graphs (refer to Figure 1). Consequently, in the overall FG decoder cycles between both sub-graphs appear, degrading in this way the performance of sum-product algorithm. However, in the proposed scheme, these cycles do not occur since in this case the sources are memoryless and non-uniform. The contribution of the sources sub-graphs is just to introduce the a priori probabilities of the non-uniform sources into the variable nodes of the corresponding RCM-LDGM factor sub-graphs.

Let us now consider the HMM source $S_{3}$ with entropy rate $ℋ(S_{3})=0.73$ and output probability profile as shown in Figure 3c. Note from this figure that the BW transformation of source $S_{3}$ can be approximated by 6 memoryless non-uniform sources ${\left\{T_{i}\right\}}_{i=1}^{6}$, with blocks of lengths $K_{1}\approx 9250,K_{2}\approx 5250,K_{3}\approx 3000,K_{4}\approx 2500,K_{5}\approx 1500,K_{6}\approx$ 15,500. Some of these blocks have short lengths, which is detrimental for the performance of the corresponding RCM-LDGM codes. To solve this problem, we build larger segments ${\tilde{T}}_{i}$ that keep the same statistical properties as previous segments. In this approach, named BWT-JSC-κ, we put together κ consecutive output blocks of the BWT to form the new segments as ${\tilde{T}}_{i}^{\left(l\right)}=\left\{T_{i}^{\left(l\cdot \kappa \right)},\ldots ,T_{i}^{\left(l\cdot (\kappa +1)-1\right)}\right\}$ for i=0,1,…,λ and l∈N. This is, in fact, similar to applying the BWT to source blocks of length κ·K, but computationally it is more efficient. The RCM-LDGM codes used to the transmit these segments have the same rate as before, but in this case their input and output block lengths are scaled by κ, i.e., $\tilde{K_{i}}=\kappa \cdot K_{i}$, $\tilde{M_{i}}=\kappa \cdot M_{i}$ and $\tilde{I_{i}}=\kappa \cdot I_{i}$, i=0,1,…,λ, respectively.

As before, Figure 4c plots the PER versus SNR curves for both strategies BWT-JSC (solid curves) and NON-BWT-JSC (dashed curves). When plotting the performance of the BWT-JSC-κ approach, two different cases have been considered, κ=1 and κ=6. Please note that when κ=1 the scheme is the same as in previous MC examples. On the other hand, by concatenating 6 consecutive BWT output segments (κ=6), we force the length of smallest segment to be 9000. Notice that for κ=6 the proposed scheme outperforms the conventional approach by 2.3 dB. However, due to the bad performance of the short block-length RCM-LDGM codes, when κ=1 the performance is similar to that of the conventional approach. This clearly shows that by concatenating BWT segments the system performance improves thanks to the avoidance of blocks with short lengths.

As summarized in Table 3, the proposed scheme clearly outperforms the conventional approach, and the PER vs SNR curves are only about 3 dB away from the Shannon limits.

## 5. Conclusions

A new source-controlled coding scheme for high-throughput transmission of binary sources with memory over AWGN channels has been proposed. The proposed strategy is based on the concatenation of the BWT with rate-compatible RCM-LDGM codes. The BWT transforms the original source with memory into a set of independent non-uniform discrete memoryless binary sources, which are then separately encoded, with optimal rates, using RCM-LDGM codes. Simulations show that the proposed scheme outperforms the traditional strategy of using the FG of the source in the decoding process by up to 4.2 dB for a spectral efficiency of 7.4 binary source symbols per complex channel use and a source with entropy rate 0.57 bits per source symbol. The resulting performance lies within 3 dB of the Shannon limit.

## Figures and Tables

**Table 1:** Markov Source Parameters.

Source	Matrix A	Matrix B	Vector π	Entropy H
$S_{1}$	$a_{11}=0.90$ $a_{22}=0.70$	$b_{11}=1.0$ $b_{22}=1.0$	[0.750.25]	0.57
$S_{2}$	$a_{11}=0.80$ $a_{22}=0.50$	$b_{11}=1.0$ $b_{22}=1.0$	[0.710.29]	0.8
$S_{3}$	$a_{11}=0.90$ $a_{22}=0.90$	$b_{11}=0.5$ $b_{22}=0.995$	[0.50.5]	0.73

**Table 2:** Design parameters (refer to Section 2.3) used for sources (**a**) $S_{1}$, (**b**) $S_{2}$ and (**c**) $S_{3}$.

		D	**M**	**I**	$d_{\mathrm{LDGM}}^{\left(v\right)}$
BWT-JSC	$K_{1}$ = 9020	{1, 1, 1, 2, 2}	3705	55	5
	$K_{2}$ = 27980	{1, 1, 1, 1, 2, 2, 2, 2}	6140	100	5
NON-BWT-JSC	*K* = 37000	{2, 2, 3, 3, 4, 7}	9860	140	5
$S_{1}$					
		D	**M**	**I**	$d_{\mathrm{LDGM}}^{\left(v\right)}$
BWT-JSC	$K_{1}$ = 26500	{2, 2, 3, 3, 4, 8}	6310	110	5
	$K_{2}$ = 10500	{2, 3, 4, 7}	3490	90	5
NON-BWT-JSC	*K* = 37000	{2, 3, 4, 4, 7}	9940	60	3
$S_{2}$					
		D	**M**	**I**	$d_{\mathrm{LDGM}}^{\left(v\right)}$
BWT-JSC-κ	$K_{1}$ = 9250	{2, 3, 4, 4, 7}	3376	29	7
	$K_{2}$ = 5250	{2, 3, 4, 4, 7}	1839	19	7
	$K_{3}$ = 3000	{2, 3, 4, 4, 7}	935	37	7
	$K_{4}$ = 2500	{2, 3, 4, 4, 7}	673	35	6
	$K_{5}$ = 1500	{2, 2, 3, 3, 4, 7}	351	18	6
	$K_{6}$ = 15500	{2, 2, 2, 3, 3, 4, 4, 7, 7}	2632	56	6
NON-BWT-JSC	*K* = 37000	{2, 2, 3, 3, 4, 8}	9880	120	3
$S_{3}$					

**Table 3:** Summary of numerical results. Labels BWT-JSC and NON-BWT-JSC represent the SNR required for a PER of $10^{-3}$ with each scheme.

	Entropy Rate	Shannon Limit	BWT-JSC(-κ)		NON-BWT-JSC
$S_{1}$	0.57	12.57 dB	15.8 dB		20 dB
$S_{2}$	0.80	17.78 dB	20.9 dB		23.25 dB
			1	6	
$S_{3}$	0.73	16.15 dB	21.8 dB	19.55 dB	21.8 dB

## Abbreviations

The following abbreviations are used in this manuscript:

BWT	Burrows-Wheeler Transform
DMS	Discrete Memoryless Source
JSC	Joint Source-Channel
MC	Markov Chain
HMM	Hidden Markov Model
RCM	Rate-Compatible Modulation
LDGM	Low-Density Generator Matrix
LDPC	Low-Density Parity Check
AWGN	Additive White Gaussian Noise
QAM	Quadrature Amplitude Modulation
SNR	Signal-to-Noise Ratio
PER	Paquet Error Rate
